# Quorum Sensing Inhibition or Quenching in *Acinetobacter baumannii*: The Novel Therapeutic Strategies for New Drug Development

**DOI:** 10.3389/fmicb.2021.558003

**Published:** 2021-02-01

**Authors:** Shan Zhong, Songzhe He

**Affiliations:** ^1^Department of Acupuncture, Guilin Hospital of Traditional Chinese Medicine, Guilin, China; ^2^Department of Clinical Laboratory, Affiliated Hospital of Guilin Medical University, Guilin, China; ^3^Department of Neurology, Nanfang Hospital, Southern Medical University, Guangzhou, China

**Keywords:** *Acinetobacter baumannii*, N-acyl-homoserine lactones, quorum sensing, quorum sensing inhibition, quorum quenching, antimicrobial resistance, biofilm formation

## Abstract

*Acinetobacter baumannii* is a Gram-negative opportunistic nosocomial pathogen, which can cause ventilator-related and blood infection in critically ill patients. The resistance of *A. baumannii* clinical isolates to common antimicrobials and their tolerance to desiccation have emerged as a serious problem to public health. In the process of pathogenesis, bacteria release signals, which regulate virulence and pathogenicity-related genes. Such bacteria coordinate their virulent behavior in a cell density-dependent phenomenon called quorum sensing (QS). In contrast, the two main approaches of QS interference, quorum sensing inhibitors (QSIs) and quorum quenching (QQ) enzymes, have been developed to reduce the virulence of bacteria, thus reducing the pressure to produce bacterial drug resistance. Therefore, QSIs or QQ enzymes, which interfere with these processes, might potentially inhibit bacterial QS and ultimately biofilm formation. In this review, we aim to describe the state-of-art in the QS process in *A. baumannii* and elaborate on the use of QSIs or QQ enzymes as antimicrobial drugs in various potential sites of the QS pathway.

## Introduction

Antimicrobial resistance (AMR) has become one of the major global public health concerns, which results mostly from the selective pressure exerted by antibiotic abuse (Borges and Simoes, 2019; Lewis, 2020; Theuretzbacher et al., 2020). Worrisomely, a recent review estimates that the AMR-causing human deaths will reach 10 million by 2050 unless a global response to the problem of AMR is mounted (O’neill, 2014). However, this prediction has been challenged due to the lack of comprehensive data on the global burden of AMR (de Kraker et al., 2016). Hence, understanding of the resistance mechanisms and the regulation of genes will help us to make a correct estimation of the situation in the future, and finally effectively prevent the rapid spread of AMR.

Recent reports, based on hospital surveillance studies and data from the American Society of Infectious Diseases, have begun to refer to microbes that are mainly involved in AMR as “ESKAPE pathogens”(Tommasi et al., 2015; Micoli et al., 2018). The term “ESKAPE” encompasses six such pathogens (*Enterococcus faecium*, *Staphylococcus aureus*, *Klebsiella pneumoniae*, *Acinetobacter baumannii*, *Pseudomonas aeruginosa*, and *Enterobacter* spp.) and are capable of “escaping” from common antibacterial treatments (Boucher et al., 2009). *Acinetobacter baumannii*, a non-fermenter Gram-negative opportunistic pathogen, associated with nosocomial infection, such as pneumonia, bloodstream, and urinary tract infections (Karageorgopoulos and Falagas, 2008; Harding et al., 2018). *Acinetobacter baumannii* infections often occur in patients with prolonged hospitalization and with long-term exposition to antimicrobials, so that its multi-drug resistance to most of the clinic antibiotics (Garnacho-Montero and Timsit, 2019; Zhao et al., 2019). In recent decades, the emergence of multi- and even pan-drug resistant *A. baumannii* has brought a tremendous challenge to the infection control and treatment plans in clinical treatment (Dijkshoorn et al., 2007; Karageorgopoulos and Falagas, 2008).

As previously stated, multi-drug resistance and biofilm of *A. baumannii* increase the difficulty of clinical treatment. Besides, bacteria can monitor the changes in the number of themselves or other bacteria in the surrounding microenvironment according to the concentration of specific signal molecules. Meanwhile, cells can communicate with each other to coordinate gene expression, so as to adapt to changing environmental conditions in the form of groups. This phenomenon is called as bacterial quorum sensing (QS) in many research reports (Diggle et al., 2007; Li and Tian, 2012). *Via* secreting and receiving signal molecules, the QS system can regulate gene expression, biofilm formation, and extracellular polysaccharides, so that bacteria as a group can jointly cope with changes in the surrounding environment, resulting in adverse consequences such as drug resistance and virulence (Dong et al., 2001; Miller and Bassler, 2001). The expression of pathogenicity and virulence through the QS system roughly includes the following steps: (I) synthesizes QS signal molecules; (II) release of signal molecules to the environment; (III) sensing and binding of the signal molecules at high cell density to membrane receptors; (IV) retrieval of the receptor-signal complex from the cell and its binding to the promoter region; and (V) transcription of pathogenicity-related genes (Deng et al., 2011; Duran et al., 2016).

In the case of Gram-positive bacteria, the signal molecules of the QS system are mainly oligopeptides acting as autoinducers (AIs), while, that of Gram-negative bacteria is interceded by N-acyl-homoserine lactones (AHLs) acting as AIs (Miller and Bassler, 2001; Shaaban et al., 2019). Moreover, another kind of signal molecule is the furanosyl borate diester molecule named autoinducer 2 (AI-2), which is found in both Gram-positive bacteria and Gram-negative bacteria (Elgaml et al., 2014). A variety of biological characteristics, including the release of virulence factors, are regulated by the QS system. The QS system can upregulate pathogenic genes, but QS interference also downregulates pathogenicity to help the immune system eradicate infected pathogens (Chen et al., 2019). Recently, inhibitors of the QS process, also called as quorum quenching (QQ) enzymes or quorum sensing inhibitors (QSIs), have been developed to reduce the virulence of bacteria, thereby inhibiting bacterial virulence factors without interfering with bacterial growth, causing less Darwinian selection pressure for bacterial resistance (Maeda et al., 2012). Therefore, the present review takes an attempt to summarize the QS system involved in the biofilm formation and other virulence of *A. baumannii*. Meanwhile, it also provides the latest development of QSIs or QQ enzymes as a possible strategy for the design of new antimicrobial agents.

## Mechanism of Quorum Sensing in *Acinetobacter baumannii*

The typical AHL system of Gram-negative bacteria is regulated by LuxI and LuxR protein families. According to reports, the LuxI-LuxR type regulatory system binds to a specific promoter sequence called lux-box, which regulates the expression of QS target genes (Egland and Greenberg, 2001; Bhargava et al., 2010). It has been reported that even though the AbaI promoter has not yet been identified, a putative *lux*-box (CTGTAAATTCTTACAG) for *A. baumannii* is located at the 67 bp upstream of the putative ATG start for AbaI and may represent the binding site of AbaR (Niu et al., 2008). Furthermore, there is a close similarity between AbaI protein and members of the LuxI family of *V. fischeri* (Milton, 2006). The protein sequence of AbaI is 27.5% identical and 46% similar to LasI of *P. aeruginosa* (Bhargava et al., 2012). Interestingly, the product of this *abaI* gene is the AHL, which has been demonstrated to be necessary for biofilm formation in *A. baumannii* (He et al., 2015).

QS system is mainly composed of AbaI, AbaR, and AHL in *A. baumannii*. The recently completed genomic sequence of *A. baumannii* ATCC17978 suggested that autoinduction synthase AbaI and acyltransferase may be the sole participants in the biosynthesis of AHL signals with different strand lengths (Niu et al., 2008). Apart from this, in a recent report, nine acinetobacter strains from patients and hospital environment were analyzed for QS signal production, they found that all members of the so-called *A. calcoaceticus-A. baumannii* complex could secret medium- to long-chain AHL (C_6_-C_14_), instead of short-chain AHL (C_4_-C_6_; Bitrian et al., 2012). Interestingly, there is evidence that 63% of *Acinetobacter* strains produced more than one AHL, but no AHL signal can be specifically assigned to specific species of the genus, indicating quorum sensors in *Acinetobacter* are not homogenously distributed among species (Gonzalez et al., 2009).

In *A. baumannii*, recent studies have linked biofilm development with QS (Kroger et al., 2016; Paluch et al., 2020; Saipriya et al., 2020). *A. baumannii* encodes homologs genes (*abaI* and *abaR*) of *Vibrio*’s archetypal QS genes *luxI* and *luxR*, respectively. Deleting *abaI* reduces biofilm formation in *A. baumannii* (Niu et al., 2008). Another significant factor that helps *A. baumannii* to produce biofilm is the production of the exopolysaccharide poly-β-1,6-N-acetylglucosamine (PNAG), which is essential for adhesion and aggregation (Choi et al., 2009). Because of this, Raorane et al. (2019) investigated the antibiofilm activities of 12 flavonoids and showed that curcumin and other flavonoids have the potential to control biofilm formation and virulence in *A. baumannii*. Similarly, according to the latest research report, four FDA-approved drugs (erythromycin, levamisole, chloroquine, and propranolol) were studied for the first time as inhibitors for QS against clinical *A. baumannii* (Seleem et al., 2020). This study showed that antibiotics like erythromycin not only had antibacterial activity but also inhibited the formation of biofilm induced by QS. This suggests that the use of FDA-approved drugs to inhibit QS is a promising strategy that can inhibit virulence without affecting the growth of microorganisms and may help to reduce the selection pressure that leads to the development of antibiotic resistance. Fortunately, Liu et al. (2020) found that after treatment with antimicrobial peptide Cec4, multiple metabolic pathways, two-component regulatory systems, quorum sensing, and antibiotic synthesis-related pathways in the biofilm of planktonic clinical carbapenem-resistant *A. baumannii* (CRAB) were affected. However, there are great differences in biofilm formation of clinical CRAB. It is reported that, although drug-resistant strains produce fragile biofilms, they still have a high level of biofilm-specific resistance (Qi et al., 2016). Therefore, deeper explorations of epidemiological studies (i.e., bacterial molecular typing, drug resistance, and virulence factor detection of clinical strains), would help us to better improve the understanding of their relationship.

Moreover, oxidative stress is also induced during the drying period in *A. baumannii* (Gayoso et al., 2014; Harding et al., 2018). In fact, it has been reported that in response to oxidative stress, the emergence of *A. baumannii* contains an insertion sequence element, IS*Aba1*, upstream of the catalase **[G]** gene, *katG*, which drives the expression of *katG* and enhances resistance to increased levels of hydrogen peroxide (Wright et al., 2017). For this reason, Bhargava et al. studied the relationship between oxidative stress and QS and reported for the first time that catalase and superoxide dismutase in *A. baumannii* are regulated by the QS system. At the same time, under the co-infection of *A. baumannii* and *P. aeruginosa*, it was found that pyocyanin, produced by *P. aeruginosa*, could induce the protective mechanism of *A. baumannii* against oxidative stress and also increase its tolerance to antibiotics, and eventually lead to hold serious implications in disease management (Bhargava et al., 2014). Therefore, future research will seek the combination of QQ and ROS generating agents like hydrogen peroxide, which may effectively control *A. baumannii* that can persist for a long time in the hospital environment.

## Quorum Sensing Inhibition in *Acinetobacter baumannii*

As is known to all, QS is a form of cell-cell communication that regulates gene expression in response to population density to coordinate collective behaviors (Gao et al., 2014). However, bacteria that can recognize this QS communication have developed the ability to interfere with it at different stages. It has been found that the QS system can be interfered in a variety of ways, roughly in the following four ways (Geske et al., 2008; Defoirdt et al., 2013; Jiang and Li, 2013; Kalia, 2013; Scutera et al., 2014; Borges and Simoes, 2019): (I) suppression of the synthesis of signal molecules; (II) enzymatically degrading signal molecules; (III) competing with signal molecules for binding to receptor sites; and (IV) interfering with the binding of signal molecules to gene promoters and inhibiting gene expression. Recently, many types of QSIs have been reported, which can be synthetic or found in nature from terrestrial, marine, or freshwater ecosystems (Kalia et al., 2019). In nature, QSIs are produced by a wide range of living organisms, such as plants, animals, fungi, or bacteria (Bjarnsholt et al., 2010; Kalia and Purohit, 2011; Moore et al., 2015; Silva et al., 2016). Most known QSIs are mainly identified in plants and bacteria. This may be due to more screening of these activities by plant extracts and bacteria (Grandclement et al., 2016; Haque et al., 2019; Mulat et al., 2019).

At present, Saroj and Rather (2013) found the QSIs potential of streptomycin at the subinhibitory concentration in *A. baumannii*, suggesting that the sub minimal inhibitory concentration (sub-MIC) of streptomycin may act as an antagonist of 3-OH-C12-HSL, interfering with the signal binding to AbaR protein. A library screening of AHLs analogs showed that non-natural ligands contained aromatic acyl groups that can block AbaR, thus inhibiting the formation of biofilm in *A. baumannii* (Stacy et al., 2012). Furthermore, Alves et al. (2016) studied the effect of linalool on the plankton cells and biofilms of *A. baumannii* on different surfaces, as well as its effect on adhesion and QS was evaluated. The results showed that linalool could inhibit the formation of biofilm of *A. baumannii*, change the adhesion of *A. baumannii* to the surface, and interfere with the QS system. Therefore, linalool may be a promising antibacterial agent to inhibit the planktonic cells and biofilms of *A. baumannii*. Altogether, it can be seen that there is great room for development to deal with the problem of drug resistance and infection of *A. baumannii via* QSIs.

In addition, there is evidence that some biological extracts or natural products may have the potential to inhibit biofilm formation and QS. The latest study found that a marine steroid Siphonocholin (Syph-1) isolated from *siphonella* can inhibit the biofilm and pellicle formation in *A. baumannii* and has anti-QS properties (Alam et al., 2020). Further, detailed *in vivo* toxicological studies are needed for the potential target of Syph-1 as a therapeutic agent. Moreover, it has been reported that activity-guided partially purified fraction (F1) from *Glycyrrhiza glabra* led to a significant reduction in QS-mediated virulence of *A. baumannii* and reduced the levels of 3-OH-C12-HSL by downregulating the expression of abaI (Bhargava et al., 2015). Similarly, Khan et al. (2018) selected nine plants from the Sudhnoti ethnopharmacological tradition used for the treatment of infectious and inflammatory disease to evaluate the *in vitro* anti-infective potential of extracts from these species against multidrug-resistant ESKAPE. The ethanolic extract of *Martynia annua* was the extract to exhibit an IC50 against *A. baumannii* (CDC-33) and possessed a certain anti-QS activity. Unfortunately, none of the extracts inhibited biofilm formation at sub-inhibitory concentrations for growth. Further studies are warranted to determine the QSIs activity by pure compounds from biological extracts or natural products to realize their actual therapeutic potential.

## Quorum-Quenching Modulation in *Acinetobacter baumannii*

To gain benefits and compete for space, nutrition, and ecological niches, microorganisms have developed many survival strategies. One of them, QS interruptions, is simple because bacteria that produce QQ enzymes can inhibit the QS regulatory behavior of competing species, thereby benefiting or avoiding being killed. Generally, QSIs (non-enzymatic methods) induce synthase or receptor inactivation *via* competitive binding, whereas QQ enzymes (enzymatic methods) switch off signal transduction through the degradation of signal molecules (LaSarre and Federle, 2013; Tang et al., 2015). The QQ mechanism can effectively interfere with any key process in QS, which may be exploited to quench QS and prevent microbial infection (inhibition of motility and biofilm formation; Dong et al., 2007).

The AHL synthase is the key enzyme in the synthesis of the signal molecules, AHLs. In *A. baumannii*, AHLs bind to receptor molecules on the cell surface and initiates the QS process. Targeting AHL synthase may be an effective QQ strategy. When the synthase is inhibited, the signal molecules are not synthesized, and hence the QS mechanism is ceased. Moreover, it may also affect biofilm formation and the virulence mechanism of cells (Lopez et al., 2018; Paluch et al., 2020). In addition, as an AHL acylase, AmiE, which hydrolyzes the amide bond of AHL, has recently been identified in *Acinetobacter* sp. strain Ooi24. Furthermore, the QQ enzyme can also be achieved through the enzymatic hydrolysis of AHL molecules by AHL lactone. Microarray analysis showed that previously cultured Ab1 (*A. baumannii* ST-2_clon_2010) in the presence of 3-oxo-C12-HSL (a QS signaling molecule) revealed a putative QQ enzyme (α/β hydrolase gene, AidA), could contribute in bacterial competition, as it is capable of hydrolyzing the signaling molecules mediated between species (Lopez et al., 2017). The newly discovered QQ enzyme MomL can effectively degrade different AHLs of various Gram-negative bacteria. It has been proved that MomL reduced biofilm formation and increased biofilm susceptibility to different antibiotics in *A. baumannii* (Zhang et al., 2017). It is worth mentioning that, Mayer et al. (2020) found that the combined action of QQ enzyme Aii20J and DNase could reduce the biofilm formation of *A. baumannii* ATCC® 17978™, indicating that QQ strategy combined with other enzyme treatment methods, such as DNase, could represent an alternative approach to prevent the colonization and survival of the pathogen on the surface and to treat of infections caused by this pathogen.

As far as the QQ system is concerned, targeting the receptor by reducing the expression of regulatory genes or regulating the activity of *abaR* will eventually contribute to the invalid binding of AHLs, thus quenching the QS system. Recent a study has detected the anti-biofilm activity of monounsaturated chain fatty acids, palmitoleic acid (POA), and myristic acid (MOA) and found that 0.02 mg/ml POA and MOA can decrease *A. baumannii* ATCC 17978 biofilm formation up to 38 and 24%, respectively, presenting a biofilm dispersing effect and drastically reduced motility. These fatty acids decreased the expression of the regulator from the LuxIR-type QS communication system AbaIR, thereby reducing the production of AHL (Nicol et al., 2018).

## Conclusion

**Figure 1:**
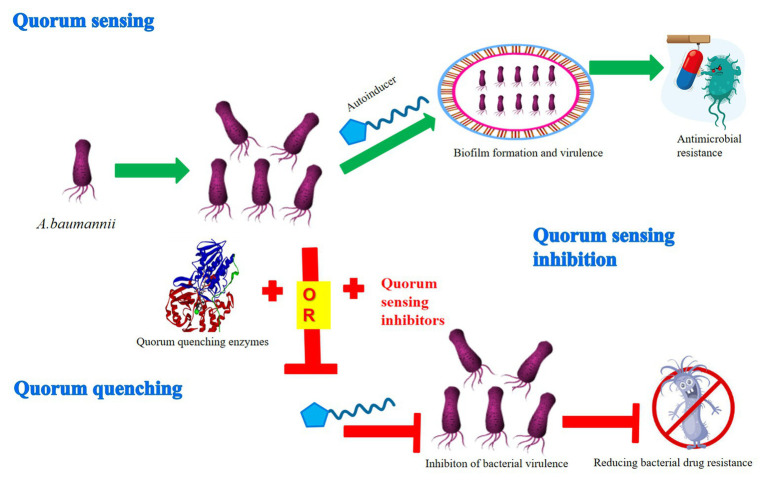
Mechanism diagram of quorum sensing inhibition or quenching through the QS system in *A. baumannii*. The QS system participates in the formation of biofilm and virulence of *A. baumannii via* various autoinducers, thus resulting in antimicrobial resistance. In contrast, the two main mechanisms of QS interference, quorum sensing inhibition and quorum quenching, have been developed to inhibit the virulence of bacteria, thus reducing the pressure to produce bacterial drug resistance.

Previous research has demonstrated that *A. baumannii* owned a strong ability to form biofilms and rapidly develop antibiotic resistance, thus it was difficult for clinicians and health care providers to treat and control its spread resulting in death (Huang et al., 2014; de Breij et al., 2018; Harding et al., 2018). In every case, the QS system confers on bacteria the ability to communicate and to change behavior in response to the presence of other bacteria. However, the evidence is accumulating that bacteria may become resistant to QSIs and QQ compounds, even without the use of QSIs and QQ compounds (i.e., when bacteria are faced with antibiotics and mutations in efflux pumps; Maeda et al., 2012; Garcia-Contreras et al., 2013; Kalia et al., 2014). Therefore, it is necessary to adopt innovative and novel strategies to expand the range of QSIs and QQ compounds against multidrug-resistant organisms. Hopefully, even with resistance arising, QSIs and QQ compounds can be used in combination with other antimicrobials (Figure 1; Dobretsov et al., 2009). Moreover, such drugs also usually do not pose an unnecessary burden on the metabolic mechanism of bacteria.

Inhibition of QS signals, which further regulates biofilm production and other possible virulence genes, has become the goal of many new therapies in recent years (Shaaban et al., 2019; Saipriya et al., 2020). Misregulation or inhibition of QS can be achieved by plant extracts (Taganna et al., 2011), synthetic compounds (Blocher et al., 2018), or AHL-degrading enzymes (Kim et al., 2014). However, most studies on QS inhibition are carried out *in vitro* and laboratory conditions using basically domesticated strains, which is a limitation. In order to counteract this effect, field investigations (*in vivo* studies) need to be carried out under the condition of simulating “real” infection (Bzdrenga et al., 2017; Chen et al., 2019). Besides, the lack of standardized methods for screening new QSIs candidates as well as the limited knowledge on the specificity of the identified QSIs continue to be a drawback, thus remaining to be explored.

QQ enzymes can be achieved by the enzymatic hydrolysis of the quorum signal by an AHL lactonase (AHLase; Dong et al., 2001). Although it has been proved that AHLases can reduce the expression level of virulence factors in *P. aeruginosa*, there is no evidence that QQ enzymes can effectively destroy the biofilm formation of bacterial pathogens (Ng et al., 2011). A previous study has shown the application of recombinant QQ enzymes in the destruction of biofilm formation by *A. baumannii* (Chow et al., 2014). These data suggest that illustrates the utility of QQ enzymes in addressing the increasing therapeutic needs of our generation.

In summary, understanding the QS of *A. baumannii* and its possible role in virulence will help to discover new biomolecules targeting the QS network to control infection. At the same time, the future development of QSIs or QQ enzymes may delay or eliminate bacterial drug resistance, laying a clinical foundation for the treatment of bacterial diseases.

## Author Contributions

All authors listed have made substantial, direct and intellectual contribution to the work, and approved it for publication.

### Conflict of Interest

The authors declare that the research was conducted in the absence of any commercial or financial relationships that could be construed as a potential conflict of interest.

## References

[ref1] Alam P. Alqahtani A. S. Mabood Husain F. Tabish Rehman M. Alajmi M. F. Noman O. M. (2020). Siphonocholin isolated from red sea sponge *Siphonochalina siphonella* attenuates quorum sensing controlled virulence and biofilm formation. Saudi Pharm. J. 28, 1383–1391. 10.1016/j.jsps.2020.09.002, PMID: 33250645 PMC7679466

[ref2] Alves S. Duarte A. Sousa S. Domingues F. C. (2016). Study of the major essential oil compounds of *Coriandrum sativum* against *Acinetobacter baumannii* and the effect of linalool on adhesion, biofilms and quorum sensing. Biofouling 32, 155–165. 10.1080/08927014.2015.1133810, PMID: 26901586

[ref3] Bhargava N. Sharma P. Capalash N. (2010). Quorum sensing in *Acinetobacter*: an emerging pathogen. Crit. Rev. Microbiol. 36, 349–360. 10.3109/1040841X.2010.512269, PMID: 20846031

[ref4] Bhargava N. Sharma P. Capalash N. (2012). N-acyl homoserine lactone mediated interspecies interactions between *A. baumannii* and *P. aeruginosa*. Biofouling 28, 813–822. 10.1080/08927014.2012.714372, PMID: 22867087

[ref5] Bhargava N. Sharma P. Capalash N. (2014). Pyocyanin stimulates quorum sensing-mediated tolerance to oxidative stress and increases persister cell populations in *Acinetobacter baumannii*. Infect. Immun. 82, 3417–3425. 10.1128/IAI.01600-14, PMID: 24891106 PMC4136213

[ref6] Bhargava N. Singh S. P. Sharma A. Sharma P. Capalash N. (2015). Attenuation of quorum sensing-mediated virulence of *Acinetobacter baumannii* by *Glycyrrhiza glabra* flavonoids. Future Microbiol. 10, 1953–1968. 10.2217/fmb.15.107, PMID: 26582430

[ref7] Bitrian M. Solari C. M. Gonzalez R. H. Nudel C. B. (2012). Identification of virulence markers in clinically relevant strains of *Acinetobacter* genospecies. Int. Microbiol. 15, 79–88. 10.2436/20.1501.01.161, PMID: 22847269

[ref8] Bjarnsholt T. Van Gennip M. Jakobsen T. H. Christensen L. D. Jensen P. O. Givskov M. (2010). *In vitro* screens for quorum sensing inhibitors and *in vivo* confirmation of their effect. Nat. Protoc. 5, 282–293. 10.1038/nprot.2009.205, PMID: 20134428

[ref9] Blocher R. Rodarte Ramirez A. Castro-Escarpulli G. Curiel-Quesada E. Reyes-Arellano A. (2018). Design, synthesis, and evaluation of alkyl-quinoxalin-2(1H)-one derivatives as anti-quorum sensing molecules, inhibiting biofilm formation in *Aeromonas caviae* Sch3. Molecules 23:3075. 10.3390/molecules23123075, PMID: 30477243 PMC6321446

[ref10] Borges A. Simoes M. (2019). Quorum sensing inhibition by marine bacteria. Mar. Drugs 17:427. 10.3390/md17070427, PMID: 31340463 PMC6669520

[ref11] Boucher H. W. Talbot G. H. Bradley J. S. Edwards J. E. Gilbert D. Rice L. B.. (2009). Bad bugs, no drugs: no *ESKAPE*! An update from the Infectious Diseases Society of America. Clin. Infect. Dis. 48, 1–12. 10.1086/595011, PMID: 19035777

[ref12] Bzdrenga J. Daude D. Remy B. Jacquet P. Plener L. Elias M.. (2017). Biotechnological applications of quorum quenching enzymes. Chem. Biol. Interact. 267, 104–115. 10.1016/j.cbi.2016.05.028, PMID: 27223408

[ref13] Chen J. Wang B. Lu Y. Guo Y. Sun J. Wei B.. (2019). Quorum sensing inhibitors from marine microorganisms and their synthetic derivatives. Mar. Drugs 17:80. 10.3390/md17020080, PMID: 30696031 PMC6409935

[ref14] Choi A. Slamti L. Avci F. Pier G. Maira-Litrán T. (2009). The *pgaABCD* locus of *Acinetobacter baumannii* encodes the production of poly-beta-1-6-N-acetylglucosamine, which is critical for biofilm formation. J. Bacteriol. 191, 5953–5963. 10.1128/JB.00647-09, PMID: 19633088 PMC2747904

[ref15] Chow J. Y. Yang Y. Tay S. B. Chua K. L. Yew W. S. (2014). Disruption of biofilm formation by the human pathogen *Acinetobacter baumannii* using engineered quorum-quenching lactonases. Antimicrob. Agents Chemother. 58, 1802–1805. 10.1128/AAC.02410-13, PMID: 24379199 PMC3957888

[ref16] de Breij A. Riool M. Cordfunke R. A. Malanovic N. De Boer L. Koning R. I.. (2018). The antimicrobial peptide SAAP-148 combats drug-resistant bacteria and biofilms. Sci. Transl. Med. 10:eaan4044. 10.1126/scitranslmed.aan4044, PMID: 29321257

[ref18] Defoirdt T. Brackman G. Coenye T. (2013). Quorum sensing inhibitors: how strong is the evidence? Trends Microbiol. 21, 619–624. 10.1016/j.tim.2013.09.006, PMID: 24126008

[ref17] de Kraker M. E. Stewardson A. J. Harbarth S. (2016). Will 10 million people die a year due to antimicrobial resistance by 2050? PLoS Med. 13:e1002184. 10.1371/journal.pmed.1002184, PMID: 27898664 PMC5127510

[ref19] Deng Y. Wu J. Tao F. Zhang L. H. (2011). Listening to a new language: DSF-based quorum sensing in Gram-negative bacteria. Chem. Rev. 111, 160–173. 10.1021/cr100354f, PMID: 21166386

[ref20] Diggle S. P. Crusz S. A. Camara M. (2007). Quorum sensing. Curr. Biol. 17, R907–R910. 10.1016/j.cub.2007.08.045, PMID: 17983563

[ref21] Dijkshoorn L. Nemec A. Seifert H. (2007). An increasing threat in hospitals: multidrug-resistant *Acinetobacter baumannii*. Nat. Rev. Microbiol. 5, 939–951. 10.1038/nrmicro1789, PMID: 18007677

[ref22] Dobretsov S. Teplitski M. Paul V. (2009). Mini-review: quorum sensing in the marine environment and its relationship to biofouling. Biofouling 25, 413–427. 10.1080/08927010902853516, PMID: 19306145

[ref23] Dong Y. H. Wang L. H. Xu J. L. Zhang H. B. Zhang X. F. Zhang L. H. (2001). Quenching quorum-sensing-dependent bacterial infection by an N-acyl homoserine lactonase. Nature 411, 813–817. 10.1038/35081101, PMID: 11459062

[ref24] Dong Y. H. Wang L. Y. Zhang L. H. (2007). Quorum-quenching microbial infections: mechanisms and implications. Philos. Trans. R. Soc. Lond. Ser. B Biol. Sci. 362, 1201–1211. 10.1098/rstb.2007.2045, PMID: 17360274 PMC2435583

[ref25] Duran N. Justo G. Z. Duran M. Brocchi M. Cordi L. Tasic L.. (2016). Advances in Chromobacterium violaceum and properties of violacein-its main secondary metabolite: a review. Biotechnol. Adv. 34, 1030–1045. 10.1016/j.biotechadv.2016.06.003, PMID: 27288924

[ref26] Egland K. A. Greenberg E. P. (2001). Quorum sensing in *Vibrio fischeri*: analysis of the *LuxR* DNA binding region by alanine-scanning mutagenesis. J. Bacteriol. 183, 382–386. 10.1128/JB.183.1.382-386.2001, PMID: 11114939 PMC94888

[ref27] Elgaml A. Higaki K. Miyoshi S. (2014). Effects of temperature, growth phase and *luxO*-disruption on regulation systems of toxin production in *Vibrio vulnificus* strain L-180, a human clinical isolate. World J. Microbiol. Biotechnol. 30, 681–691. 10.1007/s11274-013-1501-3, PMID: 24068537

[ref28] Gao J. Ma A. Zhuang X. Zhuang G. (2014). An N-acyl homoserine lactone synthase in the ammonia-oxidizing bacterium *Nitrosospira multiformis*. Appl. Environ. Microbiol. 80, 951–958. 10.1128/AEM.03361-13, PMID: 24271173 PMC3911186

[ref29] Garcia-Contreras R. Maeda T. Wood T. K. (2013). Resistance to quorum-quenching compounds. Appl. Environ. Microbiol. 79, 6840–6846. 10.1128/AEM.02378-13, PMID: 24014536 PMC3811534

[ref30] Garnacho-Montero J. Timsit J. F. (2019). Managing *Acinetobacter baumannii* infections. Curr. Opin. Infect. Dis. 32, 69–76. 10.1097/QCO.0000000000000518, PMID: 30520737

[ref31] Gayoso C. M. Mateos J. Mendez J. A. Fernandez-Puente P. Rumbo C. Tomas M.. (2014). Molecular mechanisms involved in the response to desiccation stress and persistence in *Acinetobacter baumannii*. J. Proteome Res. 13, 460–476. 10.1021/pr400603f, PMID: 24299215

[ref32] Geske G. D. O’neill J. C. Blackwell H. E. (2008). Expanding dialogues: from natural autoinducers to non-natural analogues that modulate quorum sensing in Gram-negative bacteria. Chem. Soc. Rev. 37, 1432–1447. 10.1039/b703021p, PMID: 18568169 PMC2590586

[ref33] Gonzalez R. H. Dijkshoorn L. Van Den Barselaar M. Nudel C. (2009). Quorum sensing signal profile of *Acinetobacter* strains from nosocomial and environmental sources. Rev. Argent. Microbiol. 41, 73–78. PMID: 19623895

[ref34] Grandclement C. Tannieres M. Morera S. Dessaux Y. Faure D. (2016). Quorum quenching: role in nature and applied developments. FEMS Microbiol. Rev. 40, 86–116. 10.1093/femsre/fuv038, PMID: 26432822

[ref35] Haque S. Yadav D. K. Bisht S. C. Yadav N. Singh V. Dubey K. K.. (2019). Quorum sensing pathways in Gram-positive and -negative bacteria: potential of their interruption in abating drug resistance. J. Chemother. 31, 161–187. 10.1080/1120009X.2019.1599175, PMID: 31007147

[ref36] Harding C. M. Hennon S. W. Feldman M. F. (2018). Uncovering the mechanisms of *Acinetobacter baumannii* virulence. Nat. Rev. Microbiol. 16, 91–102. 10.1038/nrmicro.2017.148, PMID: 29249812 PMC6571207

[ref37] He X. Lu F. Yuan F. Jiang D. Zhao P. Zhu J.. (2015). Biofilm formation caused by clinical *Acinetobacter baumannii* isolates is associated with overexpression of the *AdeFGH* efflux pump. Antimicrob. Agents Chemother. 59, 4817–4825. 10.1128/AAC.00877-15, PMID: 26033730 PMC4505227

[ref38] Huang G. Shen X. Gong Y. Dong Z. Zhao X. Shen W.. (2014). Antibacterial properties of *Acinetobacter baumannii* phage Abp1 endolysin (PlyAB1). BMC Infect. Dis. 14:681. 10.1186/s12879-014-0681-2, PMID: 25495514 PMC4274762

[ref39] Jiang T. Li M. (2013). Quorum sensing inhibitors: a patent review. Expert Opin. Ther. Pat. 23, 867–894. 10.1517/13543776.2013.779674, PMID: 23506025

[ref40] Kalia V. C. (2013). Quorum sensing inhibitors: an overview. Biotechnol. Adv. 31, 224–245. 10.1016/j.biotechadv.2012.10.004, PMID: 23142623

[ref41] Kalia V. C. Patel S. K. S. Kang Y. C. Lee J. K. (2019). Quorum sensing inhibitors as antipathogens: biotechnological applications. Biotechnol. Adv. 37, 68–90. 10.1016/j.biotechadv.2018.11.006, PMID: 30471318

[ref42] Kalia V. C. Purohit H. J. (2011). Quenching the quorum sensing system: potential antibacterial drug targets. Crit. Rev. Microbiol. 37, 121–140. 10.3109/1040841X.2010.532479, PMID: 21271798

[ref43] Kalia V. C. Wood T. K. Kumar P. (2014). Evolution of resistance to quorum-sensing inhibitors. Microb. Ecol. 68, 13–23. 10.1007/s00248-013-0316-y, PMID: 24194099 PMC4012018

[ref44] Karageorgopoulos D. E. Falagas M. E. (2008). Current control and treatment of multidrug-resistant *Acinetobacter baumannii* infections. Lancet Infect. Dis. 8, 751–762. 10.1016/S1473-3099(08)70279-2, PMID: 19022191

[ref45] Khan M. F. Tang H. Lyles J. T. Pineau R. Mashwani Z. U. Quave C. L. (2018). Antibacterial properties of medicinal plants from Pakistan against multidrug-resistant *ESKAPE* pathogens. Front. Pharmacol. 9:815. 10.3389/fphar.2018.00815, PMID: 30116190 PMC6082950

[ref46] Kim A. L. Park S. Y. Lee C. H. Lee C. H. Lee J. K. (2014). Quorum quenching bacteria isolated from the sludge of a wastewater treatment plant and their application for controlling biofilm formation. J. Microbiol. Biotechnol. 24, 1574–1582. 10.4014/jmb.1407.07009, PMID: 25112313

[ref47] Kroger C. Kary S. C. Schauer K. Cameron A. D. (2016). Genetic regulation of virulence and antibiotic resistance in *Acinetobacter baumannii*. Genes 8:12. 10.3390/genes8010012, PMID: 28036056 PMC5295007

[ref48] LaSarre B. Federle M. J. (2013). Exploiting quorum sensing to confuse bacterial pathogens. Microbiol. Mol. Biol. Rev. 77, 73–111. 10.1128/MMBR.00046-12, PMID: 23471618 PMC3591984

[ref49] Lewis K. (2020). The science of antibiotic discovery. Cell 181, 29–45. 10.1016/j.cell.2020.02.056, PMID: 32197064

[ref50] Li Y. H. Tian X. (2012). Quorum sensing and bacterial social interactions in biofilms. Sensors 12, 2519–2538. 10.3390/s120302519, PMID: 22736963 PMC3376616

[ref51] Liu W. Wu Z. Mao C. Guo G. Zeng Z. Fei Y.. (2020). Antimicrobial peptide Cec4 eradicates the bacteria of clinical carbapenem-resistant *Acinetobacter baumannii* biofilm. Front. Microbiol. 11:1532. 10.3389/fmicb.2020.01532, PMID: 32849322 PMC7431629

[ref52] Lopez M. Mayer C. Fernandez-Garcia L. Blasco L. Muras A. Ruiz F. M.. (2017). Quorum sensing network in clinical strains of *A. baumannii*: AidA is a new quorum quenching enzyme. PLoS One 12:e0174454. 10.1371/journal.pone.0174454, PMID: 28328989 PMC5362224

[ref53] Lopez M. Rueda A. Florido J. P. Blasco L. Fernandez-Garcia L. Trastoy R.. (2018). Evolution of the quorum network and the mobilome (plasmids and bacteriophages) in clinical strains of *Acinetobacter baumannii* during a decade. Sci. Rep. 8:2523. 10.1038/s41598-018-20847-7, PMID: 29410443 PMC5802823

[ref54] Maeda T. Garcia-Contreras R. Pu M. Sheng L. Garcia L. R. Tomas M.. (2012). Quorum quenching quandary: resistance to antivirulence compounds. ISME J. 6, 493–501. 10.1038/ismej.2011.122, PMID: 21918575 PMC3280137

[ref55] Mayer C. Muras A. Parga A. Romero M. Rumbo-Feal S. Poza M.. (2020). Quorum sensing as a target for controlling surface associated motility and biofilm formation in *Acinetobacter baumannii* ATCCⓇ 17978™. Front. Microbiol. 11:565548. 10.3389/fmicb.2020.565548, PMID: 33101239 PMC7554515

[ref56] Micoli F. Costantino P. Adamo R. (2018). Potential targets for next generation antimicrobial glycoconjugate vaccines. FEMS Microbiol. Rev. 42, 388–423. 10.1093/femsre/fuy011, PMID: 29547971 PMC5995208

[ref57] Miller M. B. Bassler B. L. (2001). Quorum sensing in bacteria. Annu. Rev. Microbiol. 55, 165–199. 10.1146/annurev.micro.55.1.165, PMID: 11544353

[ref58] Milton D. L. (2006). Quorum sensing in vibrios: complexity for diversification. Int. J. Med. Microbiol. 296, 61–71. 10.1016/j.ijmm.2006.01.044, PMID: 16487746

[ref59] Moore J. D. Rossi F. M. Welsh M. A. Nyffeler K. E. Blackwell H. E. (2015). A comparative analysis of synthetic quorum sensing modulators in *Pseudomonas aeruginosa*: new insights into mechanism, active efflux susceptibility, phenotypic response, and next-generation ligand design. J. Am. Chem. Soc. 137, 14626–14639. 10.1021/jacs.5b06728, PMID: 26491787 PMC4665086

[ref60] Mulat M. Pandita A. Khan F. (2019). Medicinal plant compounds for combating the multi-drug resistant pathogenic bacteria: a review. Curr. Pharm. Biotechnol. 20, 183–196. 10.2174/1872210513666190308133429, PMID: 30854956

[ref61] Ng F. S. Wright D. M. Seah S. Y. (2011). Characterization of a phosphotriesterase-like lactonase from *Sulfolobus solfataricus* and its immobilization for disruption of quorum sensing. Appl. Environ. Microbiol. 77, 1181–1186. 10.1128/AEM.01642-10, PMID: 21183649 PMC3067241

[ref62] Nicol M. Alexandre S. Luizet J. B. Skogman M. Jouenne T. Salcedo S. P.. (2018). Unsaturated fatty acids affect quorum sensing communication system and inhibit motility and biofilm formation of *Acinetobacter baumannii*. Int. J. Mol. Sci. 19:214. 10.3390/ijms19010214, PMID: 29320462 PMC5796163

[ref63] Niu C. Clemmer K. M. Bonomo R. A. Rather P. N. (2008). Isolation and characterization of an autoinducer synthase from *Acinetobacter baumannii*. J. Bacteriol. 190, 3386–3392. 10.1128/JB.01929-07, PMID: 18281398 PMC2347373

[ref64] O’neill J. (2014). Antimicrobial resistance: tackling a crisis for the health and wealth of nations. Rev. Antimicrob. Resist. 20, 1–16.

[ref65] Paluch E. Rewak-Soroczynska J. Jedrusik I. Mazurkiewicz E. Jermakow K. (2020). Prevention of biofilm formation by quorum quenching. Appl. Microbiol. Biotechnol. 104, 1871–1881. 10.1007/s00253-020-10349-w, PMID: 31927762 PMC7007913

[ref66] Qi L. Li H. Zhang C. Liang B. Li J. Wang L.. (2016). Relationship between antibiotic resistance, biofilm formation, and biofilm-specific resistance in *Acinetobacter baumannii*. Front. Microbiol. 7:483. 10.3389/fmicb.2016.00483, PMID: 27148178 PMC4828443

[ref67] Raorane C. J. Lee J. H. Kim Y. G. Rajasekharan S. K. Garcia-Contreras R. Lee J. (2019). Antibiofilm and antivirulence efficacies of flavonoids and curcumin against *Acinetobacter baumannii*. Front. Microbiol. 10:990. 10.3389/fmicb.2019.00990, PMID: 31134028 PMC6517519

[ref68] Saipriya K. Swathi C. H. Ratnakar K. S. Sritharan V. (2020). Quorum-sensing system in *Acinetobacter baumannii*: a potential target for new drug development. J. Appl. Microbiol. 128, 15–27. 10.1111/jam.14330, PMID: 31102552

[ref69] Saroj S. D. Rather P. N. (2013). Streptomycin inhibits quorum sensing in *Acinetobacter baumannii*. Antimicrob. Agents Chemother. 57, 1926–1929. 10.1128/AAC.02161-12, PMID: 23318804 PMC3623334

[ref70] Scutera S. Zucca M. Savoia D. (2014). Novel approaches for the design and discovery of quorum-sensing inhibitors. Expert. Opin. Drug Discov. 9, 353–366. 10.1517/17460441.2014.894974, PMID: 24597980

[ref71] Seleem N. M. Abd El Latif H. K. Shaldam M. A. El-Ganiny A. (2020). Drugs with new lease of life as quorum sensing inhibitors: for combating MDR *Acinetobacter baumannii* infections. Eur. J. Clin. Microbiol. Infect. Dis. 39, 1687–1702. 10.1007/s10096-020-03882-z, PMID: 32328851 PMC7180647

[ref72] Shaaban M. Elgaml A. Habib E. E. (2019). Biotechnological applications of quorum sensing inhibition as novel therapeutic strategies for multidrug resistant pathogens. Microb. Pathog. 127, 138–143. 10.1016/j.micpath.2018.11.043, PMID: 30503958

[ref73] Silva L. N. Zimmer K. R. Macedo A. J. Trentin D. S. (2016). Plant natural products targeting bacterial virulence factors. Chem. Rev. 116, 9162–9236. 10.1021/acs.chemrev.6b00184, PMID: 27437994

[ref74] Stacy D. M. Welsh M. A. Rather P. N. Blackwell H. E. (2012). Attenuation of quorum sensing in the pathogen *Acinetobacter baumannii* using non-native N-acyl homoserine lactones. ACS Chem. Biol. 7, 1719–1728. 10.1021/cb300351x, PMID: 22853441 PMC3477293

[ref75] Taganna J. C. Quanico J. P. Perono R. M. Amor E. C. Rivera W. L. (2011). Tannin-rich fraction from *Terminalia catappa* inhibits quorum sensing (QS) in Chromobacterium violaceum and the QS-controlled biofilm maturation and LasA staphylolytic activity in *Pseudomonas aeruginosa*. J. Ethnopharmacol. 134, 865–871. 10.1016/j.jep.2011.01.028, PMID: 21291979

[ref76] Tang K. Su Y. Brackman G. Cui F. Zhang Y. Shi X.. (2015). MomL, a novel marine-derived N-acyl homoserine lactonase from *Muricauda olearia*. Appl. Environ. Microbiol. 81, 774–782. 10.1128/AEM.02805-14, PMID: 25398866 PMC4277582

[ref77] Theuretzbacher U. Bush K. Harbarth S. Paul M. Rex J. H. Tacconelli E.. (2020). Critical analysis of antibacterial agents in clinical development. Nat. Rev. Microbiol. 18, 286–298. 10.1038/s41579-020-0340-0, PMID: 32152509

[ref78] Tommasi R. Brown D. G. Walkup G. K. Manchester J. I. Miller A. A. (2015). ESKAPEing the labyrinth of antibacterial discovery. Nat. Rev. Drug Discov. 14, 529–542. 10.1038/nrd4572, PMID: 26139286

[ref79] Wright M. S. Mountain S. Beeri K. Adams M. D. (2017). Assessment of insertion sequence mobilization as an adaptive response to oxidative stress in *Acinetobacter baumannii* using IS-seq. J. Bacteriol. 199, e00833–e008316. 10.1128/JB.00833-16, PMID: 28193905 PMC5388817

[ref80] Zhang Y. Brackman G. Coenye T. (2017). Pitfalls associated with evaluating enzymatic quorum quenching activity: the case of MomL and its effect on *Pseudomonas aeruginosa* and *Acinetobacter baumannii* biofilms. PeerJ 5:e3251. 10.7717/peerj.3251, PMID: 28462048 PMC5410158

[ref81] Zhao X. Yu Y. Zhang X. Huang B. Bai P. Xu C.. (2019). Decreased biofilm formation ability of *Acinetobacter baumannii* after spaceflight on China’s Shenzhou 11 spacecraft. MicrobiologyOpen 8:e00763. 10.1002/mbo3.763, PMID: 30379419 PMC6562233

